# Strong variations in urban allergenicity riskscapes due to poor knowledge of tree pollen allergenic potential

**DOI:** 10.1038/s41598-021-89353-7

**Published:** 2021-05-13

**Authors:** Rita Sousa-Silva, Audrey Smargiassi, Daniel Kneeshaw, Jérôme Dupras, Kate Zinszer, Alain Paquette

**Affiliations:** 1 Centre for Forest Research, Department of Biological Sciences, Université du Québec à Montréal, Montreal, QC Canada; 2 Department of Environmental and Occupational Health, School of Public Health, Université de Montréal, Montreal, QC Canada; 3 Public Health Research Institute, Université de Montréal, Montreal, QC Canada; 4 National Institute of Public Health of Quebec, Montreal, QC Canada; 5 Institut des Sciences de la Forêt Tempérée, Université du Québec en Outaouais, Ripon, QC Canada; 6 Department of Social and Preventive Medicine, Université de Montréal, Montreal, QC Canada

**Keywords:** Risk factors, Public health, Biodiversity, Ecosystem services, Urban ecology

## Abstract

Exposure to allergenic tree pollen is an increasing environmental health issue in urban areas. However, reliable, well-documented, peer-reviewed data on the allergenicity of pollen from common tree species in urban environments are lacking. Using the concept of ‘riskscape’, we present and discuss evidence on how different tree pollen allergenicity datasets shape the risk for pollen-allergy sufferers in five cities with different urban forests and population densities: Barcelona, Montreal, New York City, Paris, and Vancouver. We also evaluate how tree diversity can modify the allergenic risk of urban forests. We show that estimates of pollen exposure risk range from 1 to 74% for trees considered to be highly allergenic in the same city. This variation results from differences in the pollen allergenicity datasets, which become more pronounced when a city’s canopy is dominated by only a few species and genera. In an increasingly urbanized world, diverse urban forests offer a potentially safer strategy aimed at diluting sources of allergenic pollen until better allergenicity data is developed. Our findings highlight an urgent need for a science-based approach to guide public health and urban forest planning.

## Introduction

Trees, like all flowering plants, produce pollen in order to reproduce. The impact of pollen on human health is particularly evident in people with respiratory allergies. Proteins and glycoproteins from pollen grains can act as allergens, environmental molecules that interact with the human immune system causing allergic reactions in sensitized individuals^1^. The prevalence of allergies has increased significantly over the last few decades, particularly in urban environments^2–4^, although some studies have suggested that this trend may be reaching a plateau^5^. Nonetheless, the upward trend in allergies is a matter of concern in the context of global warming, which is expected to lengthen the growing season of plants and increase the total amount of pollen produced per season, especially for trees, even though changes are likely to be species-specific and difficult to predict^6–8^. The growing prevalence of allergies also has major economic implications, including costs associated with increased use of health services, dispensing of medication, absenteeism, and impaired performance at the workplace, with estimates in the tens of billions of dollars annually^9,10^.

Current concerns about global warming are fueling a desire for larger and more green spaces in urban areas. Planting trees has been touted as an approach to lower temperatures^11^ and energy costs^12^ while improving human health and well-being^13^. A green urban environment likely improves health through multiple pathways, such as increased psychological well-being and physical activity, greater social cohesion, and reduced exposure to air pollutants and excessive heat. However, living close to urban parks and trees may also be associated with health hazards, such as aggravating allergies through elevated exposure to allergenic pollen^14,15^.

### Are trees good or bad for respiratory diseases?

The answer to this question is twofold. On the one hand, trees can improve air quality by removing pollutants and by reducing air temperature through shading and transpiration. On the other hand, the answer to the question also depends on the species and cultivars of trees we plant and where they are planted: first, the allergen content of pollen grains varies from species to species^16,17^. Second, the trees that produce the pollen must either produce large enough quantities or be fairly widespread to produce a sufficient quantity of pollen in the air to trigger an allergic response, even in sensitized individuals^18^. Cross-reactivity to other pollen types, air pollution, and meteorological conditions can affect sensitization as well^4,19,20^. Perhaps surprisingly, we know little about the allergenic potential of many common tree species thriving in urban environments and we tend to overgeneralize from the few available studies^17,21^. The sequencing of pollen allergens, which permits the production of allergenic extracts and improves the accuracy of allergy diagnosis and treatment, is limited to 29 tree species from ten taxonomic families, and the characterization of allergens from urban trees (which number in the hundreds of species in many temperate cities in Europe and North America) remains to be done^17,22^. (The entire list of tree species can be found as Supplementary Table S1). For instance, ash (*Fraxinus* spp.) and maple trees (*Acer* spp.) currently dominate the urban forest canopy of many North American cities located in the temperate zone, but the allergenicity of their pollen remains poorly studied. Until recently, allergies to ash pollen were likely underestimated due to the absence of ash pollen-specific markers and because symptoms in individuals sensitized to ash pollen may be masked by pollen from other trees (mainly birch) that bloom at the same time of year^23,24^. Similar to ash pollen, little is known on whether specific maple species and cultivars are greater triggers of allergies than other maple species. For example, Manitoba maple trees (*A. negundo*) have been reported to elicit significant sensitization rates^25,26^, suggesting that *Acer* pollen is allergenic, but the allergenicity of pollen from other maples has not been thoroughly studied including one of the most abundant species in temperate cities of North America, Norway maple (*A. platanoides*). Similar findings have been reported for *Ginkgo biloba*: pollen-producing male ginkgo trees have been widely planted in many cities throughout the world as they adapt well to difficult growing conditions, and because female Ginkgo trees are deemed undesirable by many due to the smell of their fleshy-coated seeds during the ripening process, but the potential allergenic capacity of ginkgo pollen has not been elucidated^27^.

Notwithstanding this limited information, a number of authors have nonetheless attempted to evaluate the risk that the presence of allergenic tree species in a certain area can represent for allergic people. These estimations have been made by assigning a potential allergenic value to each species based on a defined set of parameters intrinsic to the species (e.g., allergenicity of the pollen, pollination strategy, pollination period) as well as their prevalence in a certain area (see, for example, refs.^28,29^). Although some of these parameters are measurable, such as the total amount of pollen produced, which depends on the pollination strategy (wind-pollinated species produce and release the largest amounts of pollen to offset the limited efficiency of wind as a vector of pollination^30^), others, such as the intrinsic allergenicity of the pollen grains, have been premised on assumptions of potential allergenicity as published in not peer-reviewed reports such as the OPALS (Ogren Plant Allergy Scale) system (e.g., in refs.^29,31–34^) or datasets like the Pollen.com’s pollen library (e.g., in ref.^35^). A fundamental problem with these data is that the characterization of the potential allergenicity of the tree species is heavily based on expert judgment, not allergological clinical criteria, without any documentation or well-supported scientific data for the justification of the specific allergenicity of each species. Therefore, despite the merits of accounting for parameters linked to the biology and phenology of each species as well as the number of individuals per species in any given area, indexes of the allergenic potential of urban green spaces could be expected to be strongly biased by the datasets used to designate the intrinsic allergenic potential of the pollen grains, as this information affects the final value of the allergenic risk.

Within the context that complete allergen avoidance is unrealistic and that tree pollen allergens are incompletely understood^22,36^, increasing the true diversity of the urban canopy, which accounts not only for species richness but also for the evenness of the species present, could reduce the impacts of allergenic pollen: The greater the diversity of species and the evenness of abundances among the species present, the less the likelihood of large, concentrated monospecific pollen sources^37^. For instance, the increased prevalence of plane trees (*Platanus* spp.) in many Mediterranean cities, where these trees are commonly used as ornamentals, has been inferred among the factors prompting new allergies in the population^38,39^. In this study, we focus and discuss how differing allergenicity datasets can modify ‘allergenicity riskscapes’, defined as the spatial variation in risk exposure to allergenic pollen. To illustrate the impact of these weaknesses in our knowledge of tree pollen allergenicity in characterizing the allergenicity riskscape of a city, we compared these riskscapes based on the primary allergenicity datasets available for five cities in North America and Europe. We also evaluate how, despite often contradictory reporting on tree pollen allergenicity, urban forest composition and configuration impacts the allergenic risk of urban forests.

## Results

### Tree pollen allergenicity data

Using the concept of allergenicity riskscape, we compared and contrasted the risk for pollen allergy sufferers in Barcelona, Montreal, New York City, Paris, and Vancouver, collating the information available on the allergenicity of urban trees from different datasets (see Methods). First, a total of nine independent datasets describing the allergenic potential of tree species were identified, which differ in the level of the information provided (i.e., at genus level or species level) and in the number of taxa for which data are available (Table 1). Second, an analysis of the five cities’ public tree canopy, in terms of species composition and diversity, was conducted. In total, we mapped 1,363,758 trees from 978 species and 231 genera.

**Table 1:** Description of the different data sources of tree allergenicity used in this study, including the original and redefined classes for pollen allergenicity severity (the last two columns on the right).

Dataset	Description	No. taxa	Original classification	Redefined classification
AAAAI	The *American Academy of Allergy, Asthma, and Immunology* (AAAAI) is the largest professional medical organization in the United States devoted to the allergy/immunology specialty. Resources are available at http://www.aaaai.org and include guidance to picking less allergenic plants (refs.^40^ and ^75^)	38	Highly allergenic plants	High allergenicity
			Allergenic plants	Moderate allergenicity
			Low-allergenic plants	Low allergenicity
AIA	Systematic review undertaken by Ortolani et al. (ref.^41^) and commissioned by the *Italian Association of Aerobiology* (AIA) assessing the allergy risk of over 100 plant species commonly used for urban green in Italy. For each species, a recommendation is made on the appropriateness of its use in urban green spaces, in relation to the risk of hay fever and/or allergic sensitization. The risk was assessed according to the quality of the scientific evidence and to the experts’ opinion	128	Species whose planting should be avoided	High allergenicity
			Species whose planting should be limited and/or avoided in certain locations	Moderate allergenicity
			Species whose planting has no restrictions	Low allergenicity
ARL	*Aerobiology Research Laboratories* (ARL) is a private company that monitors outdoor pollen levels in cities across Canada. Descriptions and allergenic potential of the most important pollen taxa in Canada are available at https://www.pollenexperts.ca/allergies/types-of-pollen/	25	Highly allergenic	High allergenicity
			Moderately allergenic	Moderate allergenicity
			“[I]n high enough numbers may cause allergic reactions”	Low allergenicity
Citree	The *Citree* is an urban tree database (available under: http://citree.ddns.net/index.php) supporting the selection of trees and shrubs in cities based on criteria that includes risks associated with human interaction, such as allergies. The database includes more than 350 woody species, subspecies, varieties, hybrids, and cultivars	194^a^	High allergy potential	High allergenicity
			Medium allergy potential	Moderate allergenicity
			Low allergy potential	Low allergenicity
EAN	The *European Aeroallergen Network* (EAN) gathers pollen data from several European countries and provides pollen information about pollen season and allergy risk. Public data, including profiles of allergenic plants, are available on the country pages of http://www.polleninfo.org	26	High allergenicity	High allergenicity
			Moderate allergenicity	Moderate allergenicity
			Low allergenicity	Low allergenicity
INSPQ	The National Institute of Public Health in Quebec (French: *Institut National de Santé Publique du Québec* ; INSPQ) keeps a shortlist of the main allergenic plant species in Quebec, Canada, on https://www.msss.gouv.qc.ca/professionnels/sante-environnementale/pollens/gestion-et-controle-des-autres-especes-de-pollens-allergenes/ (in French). A more comprehensive list is presented in Asselin et al. (ref.^76^)	31	***/high	High allergenicity
			**/medium	Moderate allergenicity
			*/low	Low allergenicity
OPALS	A comprehensive vegetation guide that is intended to assist people with allergies in the selection of low allergenic plants for gardening by Ogren (ref.^67^). This guide includes the *Ogren Plant Allergy Scale* (OPALS) system, which ranks over 3000 plants on a scale of 1 (least allergenic) to 10 (most allergenic)	399^a^	7–10	High allergenicity
			4–6	Moderate allergenicity
			1–3	Low allergenicity
Pollen.com	https://www.pollen.com/ is a website maintained by IQVIA™, a health care research firm. The website provides “allergy information” on more than 1200 plant species and publishes daily pollen allergy forecast maps by US state	180	Severe allergenicity	High allergenicity
			Moderate allergenicity	Moderate allergenicity
			Mild allergenicity	Low allergenicity
RNSA	The *Réseau National de Surveillance Aérobiologique* (RNSA) is the French aerobiology network in charge of collecting and analyzing airborne pollen data. A list of trees that produce allergenic pollen is available at https://www.pollens.fr/le-reseau/les-pollens. The website also provides a vegetation guide for landscaping for limited allergenic pollen emissions (ref.^68^)	75	Strong allergy potential	High allergenicity
			Medium, moderate allergy potential	Moderate allergenicity
			Low allergy potential/non-allergenic species	Low allergenicity

For each dataset, we counted the total number of taxa (reported at the species or genus level) whose allergenic potential are described therein and that occur in the studied cities. See Supplementary Table S3 for taxa-specific examples.

^a^ Includes cultivars

In general, pollen allergenicity data are provided only at the genus level (Table 1). The exceptions are the Pollen.com’s library, with pollen allergenicity descriptions for tree genus and species; and the OPALS system, whose allergenicity values are specific to genus, species, and cultivars (if they are used). However, not all of the most common genera of trees found in cities are included in all datasets and, for those that are, not all the representative species within each genus are included in each of the datasets. Data extracted from the Citree database and the systematic review by the Italian Association of Aerobiology (AIA) are provided for individual species only. These four datasets—Citree, OPALS, AIA, Pollen.com—included data for 100 or more of the species included in this study; two of them, OPALS and Pollen.com, contained data for more than two-thirds of the genera covered. These datasets covered from 99% of the genera and species in Montreal to 73% in Barcelona. For some of the genera, pollen allergen severity is identical across (almost) all datasets. For example, alder (*Alnus*) and birch (*Betula*) pollen are unanimously considered severe allergens; the European white birch (*B. pendula*) is also considered a highly-allergenic tree in all but one dataset (Pollen.com, which classifies birch trees as a moderate rather than a severe source of allergy). Yet, for many genera, the allergenicity is markedly different. For instance, in the OPALS scale, the genera *Juglans*, *Platanus*, *Quercus*, and *Ulmus* are rated as highly allergenic (e.g., *“all walnuts produce airborne pollen and allergy”*; *“elms are a major source of allergy”*); *Juglans* and *Quercus* trees are also considered to be highly allergenic by the Pollen.com’s library (*“walnut pollen was reported to be [a] serious cause of pollinosis”*; *“[oak] pollen is commonly allergenic, and may cause severe reactions”*). On the contrary, the European Aeroallergen Network (EAN) considers the allergy risk to the pollen of these four genera to be ‘low’; *Juglans* and *Ulmus* trees are also defined in the vegetation guidance by the French aerobiological monitoring network (RNSA) and the Italian Association of Aerobiology (AIA) as being of low allergenicity. Such differences in ranking also occur at the species level. Norway and silver maples (*A. platanoides* and *A. saccharinum*) are regarded as highly allergenic on the OPALS allergenic scale (8 and 7 out of 10) but the AIA’s guidance, the Citree’s, and the Pollen.com’s libraries characterize them as moderate allergens. Maples are identified as *“highly-allergenic trees”* by the AAAAI (cf. ref.^40^), but as being of low allergenicity by the European Aeroallergen Network and the National Institute of Public Health in Quebec. Ginkgo (*G. biloba*) and London plane trees (*P. acerifolia*), planted in all cities examined, are also prime examples of dissonance among the datasets. Gingko trees are listed as non-allergenic trees in the vegetation guidance of the AIA, the RNSA, and in the Pollen.com’s library, whereas in the Citree’s and OPALS’ datasets allergenicity of its pollen is recognized as high.

Among all the tree allergenicity data sources researched and analyzed, the systematic review by the Italian Association of Aerobiology (AIA; ref.^41^) stood out as the only resource with evidence-based recommendations on the appropriateness of each species’ use in urban green spaces.

### Allergenicity riskscapes

**Figure 1:**
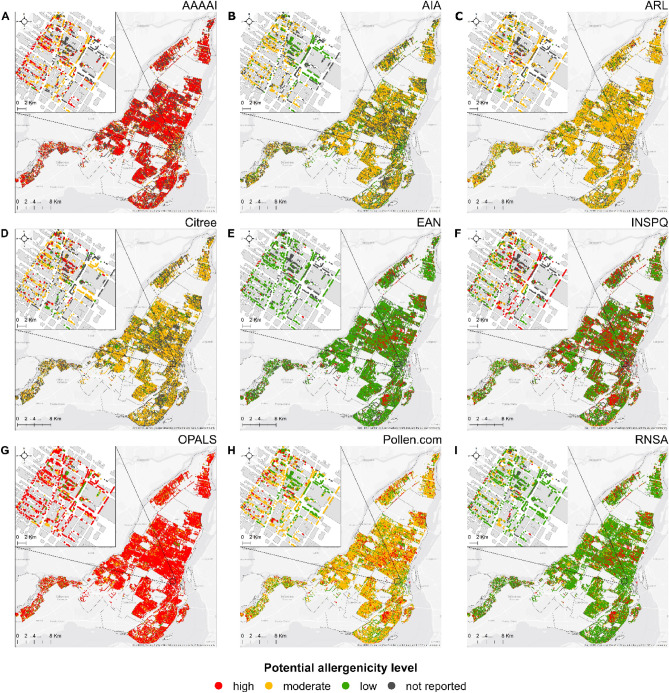
The allergenicity riskscape of the city of Montreal. The potential pollen allergenicity of each tree species within Montreal’s urban public forest was assessed using different tree allergenicity data sources, listed in alphabetical order: (**A**) the American Academy of Allergy, Asthma & Immunology (AAAAI); (**B**) the vegetation guidance by the Italian Association of Aerobiology (AIA); (**C**) Canada's Aerobiology Research Laboratories (ARL); (**D**) the Citree’s library; (**E**) the European Aeroallergen Network (EAN); (**F**) the National Institute of Public Health in Quebec (INSPQ); (**G**) the Ogren Plant Allergy Scale (OPALS); (**H**) the Pollen.com’s library; and (**I**) the vegetation guidance by the French aerobiological monitoring network (RNSA). Each dot represents one tree. The inset shows an enlarged view of a central neighborhood in the city. Data sources are presented in Table 1. Maps were created in ArcMap 10.7.1 (http://www.esri.com/).

**Figure 2:**
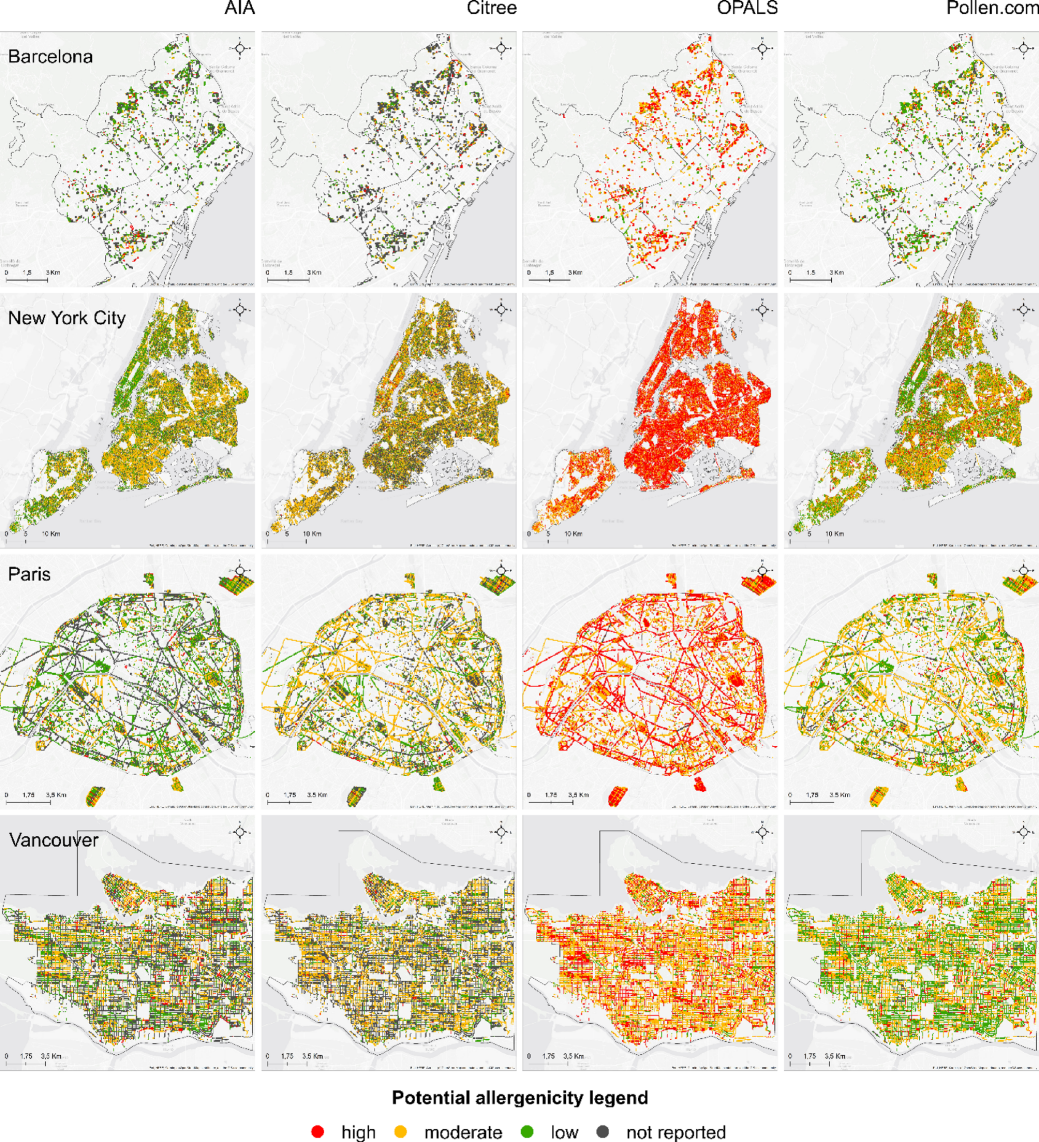
The allergenicity riskscape of the cities of Barcelona, New York City, Paris, and Vancouver based on the potential pollen allergenicity of the public trees analyzed in each city using different tree allergenicity data sources. Each dot represents one tree, each row corresponds to a single city, and each column to a different tree allergenicity data source. Only the AIA-, Citree-, OPALS-, and Pollen.com-based riskscapes are shown for presentation clarity and because the four datasets contained the largest numbers of species for which allergenicity is reported (for more than 100 species). Additional allergenicity riskscapes are provided in Supplementary Figs. S1–S4. Data sources are presented in Table 1. Maps were created in ArcMap 10.7.1 (http://www.esri.com/).

**Figure 3:**
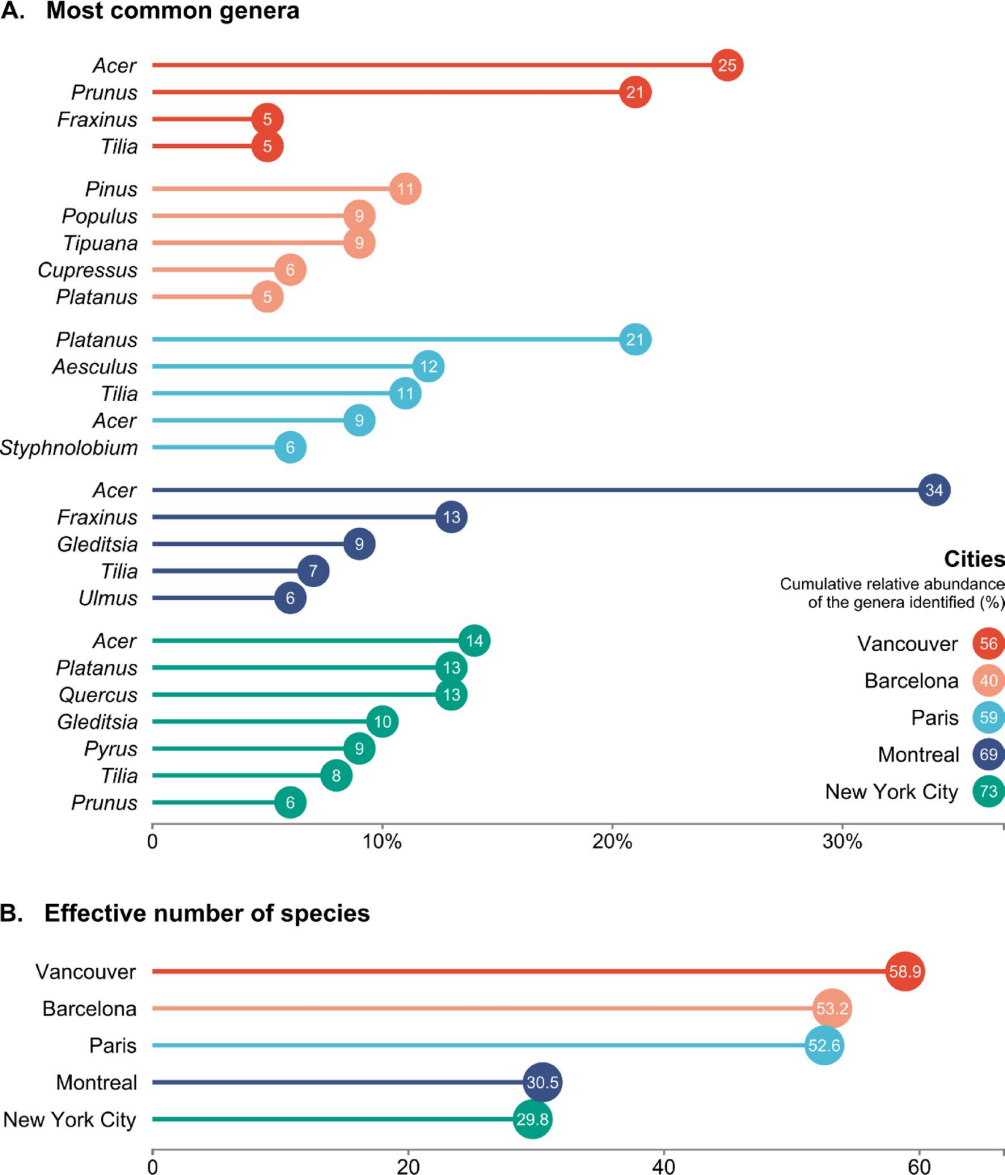
Relative abundance of the most common genera (**A**) and the effective number of species (**B**) in each of the studied cities based on the total number of public trees. For simplicity, only genera with a relative abundance greater than 5% are identified. Cities are ranked by their effective number of species, which is used as a measure of species diversity, with higher values indicating greater diversity. For more data, see Supplementary Table S2.

The resulting allergenicity riskscapes are shown in Fig. 1, in which the nine datasets are presented side-by-side for a single city, Montreal, for ease of comparison; and in Fig. 2, in which, for the four remaining cities, Barcelona, New York City, Paris, and Vancouver, only the four datasets with species-specific data—AIA, Citree, OPALS, Pollen.com—are plotted for clarity. Additional allergenicity riskscapes are provided in Supplementary Figs. S1–S4. The results show that the riskscapes changed dramatically in all cities depending upon the allergenicity dataset. In general, for all cities, the most ‘pessimistic’ scenarios (i.e., that generate a higher percentage of trees classified as highly allergenic) were based on allergenicity classifications from the OPALS and AAAAI systems, whereas the European Aeroallergen Network and the French aerobiological monitoring network datasets yielded more ‘optimistic’ estimates (Table 2). Using Montreal as an example, with the OPALS scale, 74% of the trees in Montreal are considered highly allergenic, which is three-fold higher than that of the Quebec's Institute of Public Health and 70% higher than that of the Aerobiology Research Laboratories, the organization that monitors pollen levels in cities across Canada (Table 2). That is to say, the allergenicity riskscape of the city changed depending on the dataset from predominantly high-allergenic (Fig. 1A,G), to moderate- (Fig. 1B–D,H), to low-allergenic (Fig. 1E,F,I). This finding reflects two major influences: one is the dominance of a few species and genera in the urban tree species pools (Fig. 3) and the other is that the reported allergenicity of these species and genera differ largely from one dataset to another.

**Table 2:** Percentage of trees with high, moderate, or low allergenic pollen (allergenicity severity) to the total number of public trees for each city and dataset included in the study.

	Allergenicity	Dataset								
	Severity	AAAAI	AIA	ARL	Citree	EAN	INSPQ	OPALS	Pollen.com	RNSA
Barcelona	High	14%	7%	4%	5%	1%	7%	**46%**	10%	13%
	Moderate	**32%**	16%	**21%**	**17%**	0%	**13%**	39%	25%	8%
	Low	8%	**36%**	14%	14%	**44%**	12%	14%	**38%**	**46%**
	Not reported	46%	42%	62%	65%	55%	68%	1%	27%	33%
Montreal	High	**50%**	1%	4%	3%	14%	26%	**74%**	18%	14%
	Moderate	17%	**40%**	**56%**	**45%**	0%	9%	17%	**53%**	12%
	Low	1%	20%	15%	12%	**58%**	**39%**	9%	28%	**61%**
	Not reported	32%	39%	25%	41%	28%	25%	0%	1%	14%
New York City	High	**28%**	1%	13%	3%	3%	14%	**61%**	16%	17%
	Moderate	22%	**46%**	**19%**	**58%**	0%	14%	37%	**39%**	21%
	Low	11%	30%	9%	14%	**58%**	14%	2%	36%	**42%**
	Not reported	40%	24%	58%	25%	39%	58%	0%	9%	20%
Paris	High	15%	4%	6%	2%	4%	**17%**	**49%**	7%	29%
	Moderate	**37%**	**39%**	17%	**45%**	2%	9%	46%	**51%**	16%
	Low	11%	36%	**28%**	22%	**76%**	11%	3%	32%	**46%**
	Not reported	37%	21%	49%	31%	17%	62%	1%	9%	10%
Vancouver	High	**33%**	5%	5%	2%	6%	13%	33%	10%	11%
	Moderate	19%	**28%**	**35%**	**39%**	0%	17%	**56%**	40%	15%
	Low	25%	22%	12%	13%	**68%**	**25%**	11%	**45%**	**47%**
	Not reported	24%	45%	47%	46%	26%	45%	0%	5%	27%

The ‘not reported’ category includes the trees of certain species which pollen allergenicity has not been described in the respective dataset. For each city and dataset, the most frequent severity category is highlighted in bold and coefficients greater than 50% are underlined. Percentages per city may not add up to 100% due to rounding. All numeric values are shown in Supplementary Table S4.

### Taxonomic diversity

To characterize urban tree diversity across cities, we calculated the relative abundance of all taxa present at the genus and species level (based on the total number of public trees) and used the ‘effective number of species’ to characterize and compare tree diversity between cities (see Methods). Our evaluation of tree species and genera that dominate the public tree inventories of the studied cities’ urban forests showed that *Acer* (maple) was the dominant genus in Montreal, Vancouver, and New York City, while *Platanus* (plane tree/sycamore) was the most abundant genus in Paris (over one-fifth of the total occurrences) and the second most abundant in New York City (Fig. 3 and Supplementary Table S2). In both Montreal and New York City, at the genus level, just 14 genera accounted for 90% of the total number of public trees, resulting in these cities having a low *effective* diversity, whereas in Barcelona the same cumulative abundance encompasses 31 genera. At the species level, Norway maple is fairly common in North America (Montreal, Vancouver, and New York City); and the hybrid London plane (*Platanus x acerifolia*) is ranked in the top five most abundant public tree species in three out of the five cities (Supplementary Table S2). Diversity estimates revealed that Vancouver has the highest diversity, as indicated by the effective number of species (58.91), almost twice that of Montreal (30.54) and New York City (29.76; Supplementary Table S2). Consistent with these estimates, the allergenicity riskscape of Vancouver displayed a relatively less ‘risky’ scenario than the other cities (Fig. 2 and Table 2). When looking at the four datasets with species-specific data—AIA, Citree, OPALS, Pollen.com—we observe that, according to the OPALS system, 56% of the public trees in Vancouver fall into the category of ‘moderate’ allergenic importance; whereas according to Pollen.com, the largest percentage of trees (species) fall into the categories of ‘moderate’ or ‘low’ allergenicity (85%; Table 2). These datasets included data for the overwhelming majority (more than 95%) of trees in Vancouver. Both AIA- and Citree-based maps show a similar pattern but, as these two datasets provide no data at the genus level, the picture remains largely incomplete (Fig. 2 and Table 2).

## Discussion

The different spatial patterns of human risk of exposure to allergenic pollen are of prime importance for public health and urban planning. With this knowledge, allergy sufferers could avoid high-risk areas and urban tree planners could make informed species selections. Yet importantly, reliable references for tree allergenicity are lacking. Different sources of data, for the same city and the same tree species and genera, diverge and result in very different allergenicity riskscapes. The effect of this variation is more pronounced in some of the studied cities than in others. When a city’s public tree canopy is dominated by a few common species and genera, the differences between our modeled scenarios are striking as the small number of taxa have a different allergenicity value in each dataset. The more diverse cities may be more likely to have a smaller pollen load for any given species or genus, allergenic and non-allergenic, as a high true species diversity (which reflects both the abundance and evenness of the species present) prevents the production of large quantities of monospecific pollen^37^, resulting in a lower pollen exposure risk given any allergenicity dataset.

Exposure to allergenic pollen from certain trees, grasses, and weeds is associated with a range of health effects, including allergic rhinitis, asthma, and eczema^42^. There are numerous studies that highlight urban trees as the main source of allergens in urban environments (e.g., refs.^15,29,34,35,37^), notably by trees belonging to the families Betulaceae, Fagaceae, Oleaceae, Platanaceae, and Cupressaceae^17^. However, the spectrum of tree species containing allergenic pollen is much larger than the number of species whose allergens have been identified and characterized^22^. In addition to this, for most of the tree species used in urban areas, there is no convincing evidence or only limited evidence that its pollen may be responsible for allergic sensitizations and clinically relevant allergies^26,41^. As a result, species-level or taxon information for many trees is lacking and if present, often based on assumptions and best guesses. In 2015, Ortolani et al., in their systematic review for the AIA^41^, concluded that the risk of provoking allergy was evident only for seven out of the 100 plant species assessed (and not assessable for 73 due to a lack of scientific evidence). Remarkably, there was no consensus for even one of the seven species they recommended to avoid in urban green spaces among the datasets assessed. For instance, European white birch (*B. pendula*) is considered highly allergenic by all but one dataset, suggesting that even the most evident allergenic trees can also be misused. Moreover, even though birch trees are widely recognized as allergenic trees^3,18^, they have been planted widely in cities, including some of the cities in this study. A concerted effort to reduce the presence of tree species (even if few) for which there is a consensus of high pollen allergenicity should therefore be a key objective of urban planners. In contrast, much less is known for other abundant taxa, such as *Ginkgo biloba* and *Platanus x acerifolia* trees. Their reported allergenicity ranges from low to high, but there is as yet no conclusive evidence to support either view^27,39,41,43,44^; or how the degree of allergenicity differs between species belonging to the same genus (e.g. *“Many [*Acer*] species cause allergies, but not all.*”^45^).

Given the different composition of urban forests between the five cities, it was expected that the allergenicity riskscapes would be shaped by the tree species dominating the canopy, and that is indeed what we observed. Cities with similar urban forest composition, such as Montreal and New York City, exhibit similar allergenicity riskscapes. The allergenicity riskscape of Montreal is more similar to that of New York City than to Vancouver or Paris or Barcelona. In the absence of good data on tree species allergenicity, avoiding plantations of monodominant species may be a safe ‘remedy’ for respiratory health by diluting the load of pollen of any given tree species, at any given time and location, as different species flower at different times. Moreover, increasing the diversity of tree species for which there is not consensus on allergenicity may also be relevant in promoting the development and maintenance of immune tolerance to different allergens, including those of tree pollen^46,47^.

Information on tree pollen allergenicity has two potentially distinct audiences: (i) allergy sufferers and allergologists, and (ii) urban foresters, gardeners, nursery managers, and urban planners. Regarding allergy sufferers, we are all exposed to allergens but only previously sensitized individuals develop allergic symptoms. Respiratory allergies affect approximately 10–30% of the global population, including about 8 million Canadians and 20 million Americans^9,48^. Recent estimates suggest the annual cost of allergies to the health care system and the economy in the United States to be approximately $18 billion^49^. Allergen avoidance is generally the cornerstone of the management of allergic diseases, both in preventing allergic sensitization and reducing symptoms. Pollen allergy sufferers are usually instructed to stay indoors and avoid exposure to pollen allergens as much as possible^36,50^. Pollen information services are therefore meant to provide pollen information and forecasts that help inform allergy sufferers^51^. However, pollen information is based on insufficient scientific information on the allergenicity of many tree species, the cross-reactivity between pollen from similar species, in addition to the challenges of the lack of uniformity and data completeness from relevant data sources^19,22,41^. The ease of access to an ever-growing volume of online information may further spread misinformation as inadequate or false pollen information could harm those affected with pollen allergies^51,52^.

Many cities have heavily invested in maintaining and increasing urban forest canopies, but until recently little attention has been paid to the planning of canopies with a low allergy impact. Indexes of urban green allergenicity, such as that proposed by Cariñanos et al.^29^, may provide an important step into pinpointing the potential allergenic risk that the presence of certain tree species can pose for allergic people, as they also include information on the biology and phenology of each species, such as the pollination strategy and the length of the flowering period in each area. The accuracy of the allergenic risk assessment will, however, depend on the quality of the pollen allergenicity data used as input. Planting the right tree in the right place can help reduce the harmful impact of allergenic pollen while ensuring that the benefits of green spaces for general health and well-being are maximized^37,53–55^. In recent decades, city planning strategies have promoted the masculinization of the urban forest, i.e., planting male trees (often deceitfully labeled as ‘sterile’) over female trees for their ‘litter-free’ characteristics. For instance, when the Dutch elm disease swept through North America killing millions of elm trees, the US Department of Agriculture recommended using male ash and maple trees to replace the dead elms, drastically increasing the presence of pollen in cities^56,57^. While female plants may shed unwanted seeds and fruits, male trees produce pollen. Male trees are often selected from asexually propagated clones, leading to an overabundance of certain species and cultivars that act as principal pollen sources^37^. Moreover, many of the dead elms were replaced with ash trees that are now threatened by the emerald ash borer, an invasive beetle from Asia^57^. The plight of the American elm (*Ulmus americana*) and ash trees (*Fraxinus* spp.) are examples of poor landscaping and a cautionary tale suggesting the prioritizing of tree species diversity in cities.

Taxonomic (genera and species), functional (plant size, pollen size, and number), and biological diversity (pollination strategies, flowering phenology) is key not only for preventing widespread canopy loss but may also be for minimizing the impact of highly allergenic species. Vancouver has a higher diversity, as indicated by the effective number of species, compared with the other studied cities, and fared better than the other cities under most scenarios (Table 2). Vegetation diversity may protect against respiratory allergies through greater and more diverse microbial exposure that is vital for the development of the immune system^46,47^. Importantly, the amount of pollen released is directly related to the number of trees belonging to one species. A greater diversity and evenness of tree species leads to lower concentrations of monospecific pollen at any one point in time. Insect-pollinated species may also be favored as the amount of pollen they release is negligible except in their immediate vicinity^29^. From an urban planning perspective, there is great potential for including the allergenic potential of trees as a criterion for tree selection in urban areas, which is directly related to public health goals, while ensuring appropriate diversity of species to meet resilience goals^37,58^. The first step is reliable, species-specific data (i.e., not at the genus or family levels) on the allergenicity of pollen.

The shortcomings in the current state of knowledge regarding tree pollen that cause allergies are directly reflected in the allergenicity riskscapes presented in this study, demonstrating the variation between datasets. A fundamental problem that arises from these datasets is that the descriptions of allergenicity for each species or genus are not supported by any scientific evidence or other verifiable data—with the notable exception of the AIA’s recommendations. Therefore, it is impossible to explain discrepancies between datasets or identify which scenario is the most realistic. Given this significant limitation of the data, the interpretation of a scenario as ‘pessimistic’ or ‘optimistic’ was based exclusively on the percentage of trees (species/genera) valued as being of low, moderate, or high allergenicity according to the classification obtained from each of the allergenicity datasets. Nevertheless, we acknowledge that the establishment of general allergenicity levels is not an easy task. First, because the manifestation of allergic symptoms can be triggered either by gradually cumulative exposure to certain types and certain amounts of pollen or by immediate exposure to large amounts of pollen, of the same species or those of other species^50^. Second, because the relationship between allergic symptoms and pollen abundance can differ significantly not only among different cities, countries, and bioclimatic regions, but also among different individuals within the same city, and for each different pollen type^4,20^. Given these challenges and the present incomplete understanding of tree pollen allergens and tree pollen allergies^17^, we argue that the best available option is to interpret the available data cautiously and to present them in an evidence-based manner. Implementing datasets outside of the respective region they were created for could induce errors in forecasting risks; we thus recommend that regions where the information on pollen production and allergenicity is derived from and where it should be applied to should be clearly stated, to ensure sound advice. Moving forward, we hope that the assumptions of potential allergenicity be updated in accordance with the current best evidence and continue to be updated as new data become available.

Another limitation of our study is that it does not include trees on private property, which are an important part of the urban forest, as they are not commonly included in municipal tree inventories. Consequently, the global picture of the species diversity of an urban forest remains incomplete, irrespective of the city. This is mainly due to the difficulties in performing inventories on privately owned land where access restrictions limit data collection. Although this could affect the absolute scores that we present for each city, the allergenicity riskscapes obtained with different databases of tree allergenicity would likely be similar as species selection decisions on both public and private land are constrained by nursery supply, and nurseries favor species with established demand, reinforcing private landowners decisions to plant common species^59^. Improvements in remotely sensed data (e.g., satellite imagery, LiDAR) will allow future studies to complete inventories but, at present, such data are not widely available. Other sources of bias may, however, remain as the very definition of an urban forest, which is linked to the definition of an urban area, can have different meanings depending on the country^60^. Nonetheless, from a policy perspective, an advantage of using municipal tree inventories is that those are the trees that policymakers and urban forest planners are responsible for and can act upon.

Further studies should also integrate indicators of allergy and asthma morbidity, such as medication sales and asthma-related emergency department visits, and account for the spatial variation in tree pollen concentrations within cities. Also, pollen dispersion within urban areas, in which both sources of pollen (trees) and barriers to dispersion (buildings) are present, is poorly characterized and requires more than a single or few pollen monitoring stations per city^61,62^. The pollen grains sampled at monitoring stations are rarely identified below the genus level in pollen monitoring, owing to the difficulty of differentiating species based on morphology, and therefore pollen counts often cannot be assigned to specific species^63,64^.

The cross-reactivity between pollen allergens is difficult to address given the complex interplay of co-sensitization (sensitivity to several trees, pollinating at the same time) and cross-reactivity (immune response against unrelated but similar allergenic molecules), including between tree and grass allergenic species^9^. The diagnostic accuracy of allergy testing and the efficacy of therapeutic options (avoidance, medication, immunotherapy) are also often impaired by cross-reactivity among species and sometimes families^19^. Further pollen allergen research is urgently needed to identify and characterize the molecular features of tree pollen allergens, especially those from under-studied species and genera that are abundant in urban forests. A systematic molecular classification of pollen allergens would also advance the understanding and prediction of cross-reactivity.

In conclusion, we found that the allergenicity riskscape of a city changes dramatically depending on the tree allergenicity data source. The lack of reliable, scientific-based data on tree pollen allergenicity is particularly concerning due to the long lifespan of trees, as current decisions (from homeowner preferences to urban planning choices) determine the future environmental health riskscape. As stated by Bastl et al., *“pollen information based on unreliable datasets… must be avoided by all means possible”*^51^. Importantly, the findings from this study strongly support recent calls for interdisciplinary research on urban greening and respiratory health as a means to provide meaningful public health and urban planning guidance^21,55^.

## Methods

### Tree allergenicity data

We began this study by searching for tree allergenicity datasets. Our goal was to organize the information on the allergenic potential of tree species based on data published in public health reports, health-dedicated websites, as well as reliable guidance references available to those involved in the planning and design of urban green spaces. It should be noted that we did not intend to perform a systematic collation of individual studies on tree pollen allergenicity (for reviews, see e.g., refs.^17,41^). Instead, we were interested in compiling available data from already existing databases, in which a complete set of information is presented for as many taxa as possible in a single dataset, and which are commonly and widely used in the literature (e.g., refs.^29,31–35^) and in public health reports providing guidance on low-allergenic trees that should be preferred in urban greening (e.g., refs.^65,66^). We searched in Google Scholar for studies that cited references of data sources describing the allergenic potential of tree species, using keywords such as ‘tree pollen allergy’, ‘tree pollen allergenicity’, ‘tree pollen allergens’, ‘tree allergenicity’, ‘allergenic trees’, and ‘allergic trees’, but we note that our screening was not exhaustive. Note that for the purpose of this study, we define ‘tree allergenicity’ as the potential allergenicity of the pollen from each taxon. A total of nine datasets were assessed (*AAAAI*, American Academy of Allergy, Asthma & Immunology; *AIA*, Italian Association of Aerobiology; *ARL*, Canada's Aerobiology Research Laboratories; *Citree*’s library; *EAN*, European Aeroallergen Network; *INSPQ*, Quebec's Institute of Public Health; *OPALS*, Ogren Plant Allergy Scale; *Pollen.com*’s library; *RNSA*, French aerobiological monitoring network). These sources are described in Table 1.

As the datasets analyzed were obtained from independent sources, we were interested to determine whether they would lead to similar or different results—the allergenicity riskscapes. It should be clarified that these datasets do not state their methodology and sources for establishing the criteria for assigning pollen allergenicity levels to tree species—with the noticeable exception of the study published by the AIA (ref.^41^). Given this omission, we could not assess the quality of the data. For example, the OPALS system is described as being based on the characteristics of each plant (e.g., pollen weight, size, and stickiness, sexual reproduction, pollination, and flowering); however, the inferences made by Ogren^67^ cannot be validated as these characteristics are not reported on an individual level (i.e., for each species and genus) and generalizations are made without providing evidence or references. Pollen.com and RNSA, in their species and genus factsheets, provide a short description of each taxon, but the descriptions remain vague and the statements of allergenicity are also made without justification or appropriate references (see refs.^68,69^). Furthermore, the potential allergenicity of each species is reported differently across datasets (Table 1). For instance, Citree, EAN, INSPQ, Pollen.com, and RNSA assigned the trees to three classes based on their pollen allergenic potential (albeit with different names, such as either medium or moderate potential, low or mild); AIA’s recommendations were formulated concerning their use in urban spaces, which were also grouped into three classes (allergenic plants that should be avoided, planted with limitations, or without restrictions); the OPALS scale scored each plant from 1 (least allergenic) to 10 (most allergenic). To facilitate comparisons among the different datasets, the original scores for allergenic potential were standardized into three classes: high allergenicity, moderate, and low (see Table 1). When no tree allergenicity data were available at the species (or cultivar) level, allergenicity was assigned based on classification at the genus level when the information at the genus level was provided. For taxa for which there was no information of their potential allergenicity in the datasets, we categorized them as ‘not reported' cases and they were included as such in the calculation of percentages (Table 2).

### Urban tree data

In a second step, we created an extensive dataset of urban public tree inventories by retrieving, cleaning (for typing errors and misspellings, assigning synonyms to their accepted names), and aggregating data for five major cities: Barcelona (Spain), Montreal (Canada), New York City (United States), Paris (France), and Vancouver (Canada). Montreal was the primary example in this study given the extensive inventory of trees on city property (on streets, in parks, etc.) as well as for the low evenness in the species abundances, with five species accounting for more than 50% of the diversity (Supplementary Table S2). The other four cities studied—Barcelona, New York City, Paris, and Vancouver—were included in the analyses as we wanted to compare and contrast allergenicity riskscapes, derived from different, independent datasets that describe the allergenic potential of urban forests of different composition and diversity. Vancouver, on the west coast of Canada, was selected so as to include a different tree species pool, whereas Montreal and New York City were reported to be fairly similar^70^. Barcelona and Paris were also selected to give a broad picture of the urban forests of Europe in addition to cities in North America. The urban forest definitions between these cities also differed as do human population density and potential exposure. Evaluating such a wide variation in conditions will help to ensure that consistent trends are indeed robust.

Urban public tree inventories were obtained from publicly available data repositories and are cited accordingly in Supplementary Table S5. Urban tree inventories are used as management and planning tools and include information on the tree species and the geographic location of each tree, which allows for GIS analysis, but they frequently count only trees on public land.

Species diversity was estimated using the Shannon diversity index ($H=-\sum {p}_{i}{\mathrm{ln}p}_{i}$ ; where *p* is the proportion of species ‘*i*’ in the sample), which accounts for both species abundance and evenness, and Hill numbers (exponent of Shannon’s index; ${e}^{H}$), which can be understood as the effective number of species that a community would contain if it had the same diversity but equally abundant species. The benefit of calculating the effective number of species, also called ‘true diversity’, is that it can be easily interpreted and directly compared across communities^71^. We used municipal tree inventories as input data for estimating the diversity. All data preparation and harmonization were done in R, based on packages from the 'tidyverse' family^72^. Higher taxonomy levels and standard nomenclature were obtained from the NCBI taxonomy database using the ‘taxize’ package^73^. Diversity measures were calculated using the ‘vegan’ package^74^.

Finally, we mapped the public tree canopy of each city according to the different tree pollen allergenicity datasets, where each tree identified in the cities’ tree inventories was represented as a point whose color codes for the allergenicity value. As with any research, we note that these datasets are used only as examples, meaning that implementing them outside of the respective region they were created for should be done consciously and cautiously to avoid making overreaching recommendations. High levels of allergenicity are color-coded in red, moderate levels in yellow, and low levels in green. For clarity of presentation, overlapping points (trees) were transformed into a raster layer using the majority method (i.e., by assigning each new raster cell the most frequent pixel value located within that cell). All spatial analyses were carried out using ArcMap version 10.7.1 (http://www.esri.com/).

## Supplementary Information

Supplementary Information.
