# Supplementary material for: Strong variations in urban allergenicity riskscapes due to poor knowledge of tree pollen allergenic potential
Source: Sci Rep. 2021 May 13;11:10196. doi: 10.1038/s41598-021-89353-7 (PMC8119473; doi:10.1038/s41598-021-89353-7)
Supplement: Supplementary file 1 — Supplementary Information. [file 41598_2021_89353_MOESM1_ESM.pdf]

SUPPLEMENTARY INFORMATION

Strong variations in urban allergenicity riskscapes due to poor knowledge of tree pollen allergenic potential

Rita Sousa-Silva * · Audrey Smargiassi · Daniel Kneeshaw · Jérôme Dupras · Kate Zinszer · Alain Paquette

* To whom correspondence should be addressed. Email: silva.as.rita@gmail.com.

Table S1. Taxonomic classification of all tree species documented to date having allergenic pollen with sequenced airborne allergens acknowledged by the World Health Organization and the International Union of Immunological Societies (WHO/IUIS) Allergen Nomenclature Sub-committee (www.allergen.org; release of June 1, 2020). Taxonomic names follow NCBI's classification of species.

Class	Order	Family	Genus	Species	N allergens
Magnoliopsida	Fabales	Fabaceae	*Prosopis*	*Prosopis juliflora*	2
			Vachellia	*Vachellia farnesiana*	2
	Fagales	Betulaceae	*Alnus*	*Alnus glutinosa*	2
			Betula	*Betula pendula*	7
			Carpinus	*Carpinus betulus*	1
			Corylus	*Corylus avellana*	3
			Ostrya	*Ostrya carpinifolia*	1
		Fagaceae	*Castanea*	*Castanea sativa*	1
			Fagus	*Fagus sylvatica*	1
			Quercus	*Quercus alba*	1
				Quercus ilex	1
				Quercus mongolica	1
	Gentianales	Rubiaceae	*Coffea*	*Coffea arabica*	3
	Lamiales	Oleaceae	*Fraxinus*	*Fraxinus excelsior*	1
			Ligustrum	*Ligustrum vulgare*	1
			Olea	*Olea europaea*	15
			Syringa	*Syringa vulgaris*	2
	Malpighiales	Salicaceae	*Populus*	*Populus nigra*	1
	Malvales	Malvaceae	*Triplochiton*	*Triplochiton scleroxylon*	1
	Proteales	Platanaceae	*Platanus*	*Platanus x acerifolia*	3
				Platanus orientalis	3
	Rosales	Rosaceae	*Prunus*	*Prunus persica*	1
Pinopsida	Cupressales	Cupressaceae	*Chamaecyparis*	*Chamaecyparis obtusa*	3
			Cryptomeria	*Cryptomeria japonica*	3
			Cupressus	*Cupressus arizonica*	1
				Cupressus sempervirens	4
			Juniperus	*Juniperus ashei*	4
				Juniperus oxycedrus	1
				Juniperus virginiana	2

Table S2. The five most common species across the five cities studied, including their absolute and percent abundance (relative to the total number of public trees), and the overall taxonomic diversity of the urban forests studied at order, family, genus, and species level. N, total number of taxa with a relative abundance of more than 1%; H, Shannon's diversity index; ENS, effective number of species. The most common genera are presented in Figure 3 of the main manuscript (relative abundance greater than 5%). Data sources for tree inventories are presented in Table S5.

City	Taxon				Absolute abundance	Percent abundance
	Species	Genus	Family	Order		
**Montreal**	*Acer platanoides*	*Acer*	Sapindaceae	Sapindales	50,861	16%
	Acer saccharinum	*Acer*	Sapindaceae	Sapindales	36,164	12%
	Fraxinus pennsylvanica	*Fraxinus*	Oleaceae	Lamiales	34,681	11%
	Gleditsia triacanthos	*Gleditsia*	Fabaceae	Fabales	26,827	9%
	Tilia cordata	*Tilia*	Malvaceae	Malvales	17,707	6%
					Cumulative relative abundance	54%
N	12 (out of 269)	13	10	8		
H		3.42				
ENS		30.54				
Total number of public trees		311,050				
**Vancouver**	*Prunus serrulata*	*Prunus*	Rosaceae	Rosales	13,355	9%
	Acer platanoides	*Acer*	Sapindaceae	Sapindales	11,970	8%
	Prunus cerasifera	*Prunus*	Rosaceae	Rosales	12,104	8%
	Acer rubrum	*Acer*	Sapindaceae	Sapindales	8,436	6%
	Carpinus betulus	*Carpinus*	Betulaceae	Fagales	5,190	4%
					Cumulative relative abundance	35%
N	13 (out 348)	14	9	7		
H		4.08				
ENS		58.91				
Total number of public trees		146,534				
**New York City**	*Platanus x acerifolia*	*Platanus*	Platanaceae	Proteales	87,014	13%
	Gleditsia triacanthos	*Gleditsia*	Fabaceae	Fabales	64,264	10%
	Pyrus calleryana	*Pyrus*	Rosaceae	Rosales	58,931	9%
	Quercus palustris	*Quercus*	Fagaceae	Fagales	53,185	8%
	Acer platanoides	*Acer*	Sapindaceae	Sapindales	40,112	6%
					Cumulative relative abundance	46%
N	15 (out of 131)	13	10	9		
H		3.39				
ENS		29.76				
Total number of public trees		652,169				

**Barcelona**	*Tipuana tipu*	*Tipuana*	Fabaceae	Fabales	4,030	9%
	Pinus pinea	*Pinus*	Pinaceae	Pinales	2,924	6%
	Cupressus sempervirens	*Cupressus*	Cupressaceae	Cupressales	2,409	5%
	Pinus halepensis	*Pinus*	Pinaceae	Pinales	2,249	5%
	Platanus x acerifolia	*Platanus*	Platanaceae	Proteales	2,2515	5%
				Cumulative relative abundance		50%
N	18 (out of 274)	19	14	11		
H		3.97				
ENS		53.21				
Total number of public trees		47,228				
**Paris**	*Platanus x acerifolia*	*Platanus*	Platanaceae	Proteales	36,670	18%
	Aesculus hippocastanum	*Aesculus*	Sapindaceae	Sapindales	20,035	10%
	Styphnolobium japonicum	*Sophora*	Fabaceae	Fabales	11,720	6%
	Acer pseudoplatanus	*Acer*	Sapindaceae	Sapindales	7,316	4%
	Tilia tomentosa	*Tilia*	Malvaceae	Malvales	7,614	4%
				Cumulative relative abundance		42%
N	16 (out of 690)	14	11	9		
H		3.96				
ENS		52.59				
Total number of public trees		205,232				

Table S3. Examples of different pollen allergenicity values for taxa of the genus *Fraxinus* (ash trees). This table includes the original and the redefined classes for pollen allergenicity assigned to each taxon for each of the different datasets. For the full name of each dataset, please see Table 1. Letter superscripts denote: (a) recommendation for *Fraxinus excelsior*, valid also for *F. angustifolia*, *F. ornus*, and others; translated from the original in Italian; (b) translated from the original in Italian: *Specie la cui piantagione non ha restrizioni*; (c) translated from the original in French: *Moyen*; (d) translated from the original in French: *Potentiel allergisant fort*; (f) translated from the original in French: *Espèce non allergisante*; (e) translated from the original in French: *Beaucoup*.

	**AAAAI**		**AIA**		**ARL**		**Citree**		**EAN**	
**Taxa**	*Original classification*	*Redefined*	*Original classification*	*Redefined*	*Original classification*	*Redefined*	*Original classification*	*Redefined*	*Original classification*	*Redefined*
Fraxinus	Highly-allergenic trees	High allergenicity	NA		Moderately allergenic trees	Moderate allergenicity	NA		"Allergolog-ically often overlooked."	High allergenicity
F. americana	NA		NA		NA		NA		NA	
F. angustifolia	NA		[a]	Low allergenicity	NA		NA		NA	
F. excelsior	NA		Species whose plant-ing has no restrictions [b]	Low allergenicity	NA		Medium allergy potential	Moderate allergenicity	NA	
F. excelsior 'Pendula'	NA		NA		NA		NA		NA	
F. ornus	NA		[a]	Low allergenicity	NA		NA		NA	
F. pennsylvanica	NA		NA		NA		NA		NA	

	INSPQ		OPALS		Pollen.com		RNSA	
**Taxa**	*Original classification*	*Redefined*	*Original classification*	*Redefined*	*Original classification*	*Redefined*	*Original classification*	*Redefined*
Fraxinus	Medium [c]	Moderate allergenicity	"Ash are large, native, deciduous trees that produce copious amounts of potent pollen."	High allergenicity	Moderate	Moderate allergenicity	Strong allergy potential [d]	High allergenicity
F. americana	Medium [c]	Moderate allergenicity	7	High allergenicity	Severe	High allergenicity	NA	
F. angustifolia	NA		1	Low allergenicity	NA		Non-allergenic species [e]	Low allergenicity
F. excelsior	NA		7	High allergenicity	Severe	High allergenicity	Strong allergy potential [d]	High allergenicity
F. excelsior 'Pendula'	NA		1	Low allergenicity	NA		NA	
F. ornus	NA		6	Moderate allergenicity	NA		Strong allergy potential [d]	High allergenicity
F. pennsylvanica	High [f]	High allergenicity	7	High allergenicity	Severe	High allergenicity	NA	

Table S4. Number of public trees with high, moderate, or low allergenic pollen (allergenicity severity) in each city based on the different datasets. The 'not reported' category includes the trees of certain species which pollen allergenicity has not been described in the respective dataset. For the full name of each dataset, please see Table 1.

	Allergenicity Severity	*AAAAI*	*AIA*	*ARL*	*Citree*	*EAN*	*INSPQ*	*OPALS*	*Pollen.com*	*RNSA*
Barcelona	high	6694	3232	1810	2167	380	3106	21911	4866	6157
	moderate	15134	7599	9717	7865	174	6272	18515	11635	3975
	low	3708	16771	6494	6640	20777	5777	6491	17811	21740
	not reported	21692	19626	29207	30556	25897	32073	311	12916	15356
Montreal	high	155646	2008	13420	8465	42059	81614	230883	55553	43257
	moderate	51861	125197	173132	139687	1439	28974	51714	166108	36590
	low	3283	62612	48129	36469	179995	122642	27851	87811	188206
	not reported	100260	121233	76369	126429	87557	77820	602	1578	42997
New York City	high	181890	3546	86889	21388	21428	92328	397295	105846	112801
	moderate	143081	296973	125250	376538	2154	94000	241503	252715	138529
	low	68591	194252	59525	94266	375917	90321	13371	235913	271107
	not reported	258607	157398	380505	159977	252670	375520	0	57695	129732
Paris	high	31053	7762	11692	4878	9206	34565	100066	14845	60073
	moderate	76376	80714	34778	91884	5104	18959	95335	105419	31847
	low	21677	73496	57716	45635	155459	23584	6968	65880	93391
	not reported	76126	43260	101046	62835	35463	128124	2863	19088	19921
Vancouver	high	48105	6902	8036	2251	9083	19052	48036	13943	16751
	moderate	27492	41518	51608	57260	147	24557	81682	58975	21463
	low	36485	32701	17732	18938	99487	37314	16814	66098	68589
	not reported	34452	65413	69158	68085	37817	65611	2	7518	39731

Table S5. Urban public trees inventories. All data were obtained from open data portals of the respective cities.

City	Country	Source	Year census	Last modified	Data accessed	No. trees	No. species
Barcelona	Spain	https://opendata-ajuntament.barcelona.cat/data/dataset/arbrat-zona	2018	2020	03/31/2020	47,228	273
Montreal	Canada	http://donnees.ville.montreal.qc.ca/dataset/arbres	2018	2020	03/31/2020	311,050	269
New York City	United States	https://data.cityofnewyork.us/Environment/2015-Street-Tree-Census-Tree-Data/uvpi-gqnh	2015	2016	03/31/2020	652,169	131
Paris	France	https://opendata.paris.fr/explore/dataset/les-arbres/information/	2014	2020	03/31/2020	205,232	689
Vancouver	Canada	https://opendata.vancouver.ca/explore/embed/dataset/street-trees/	2016	2020	03/31/2020	146,534	348
						1 363,758	978

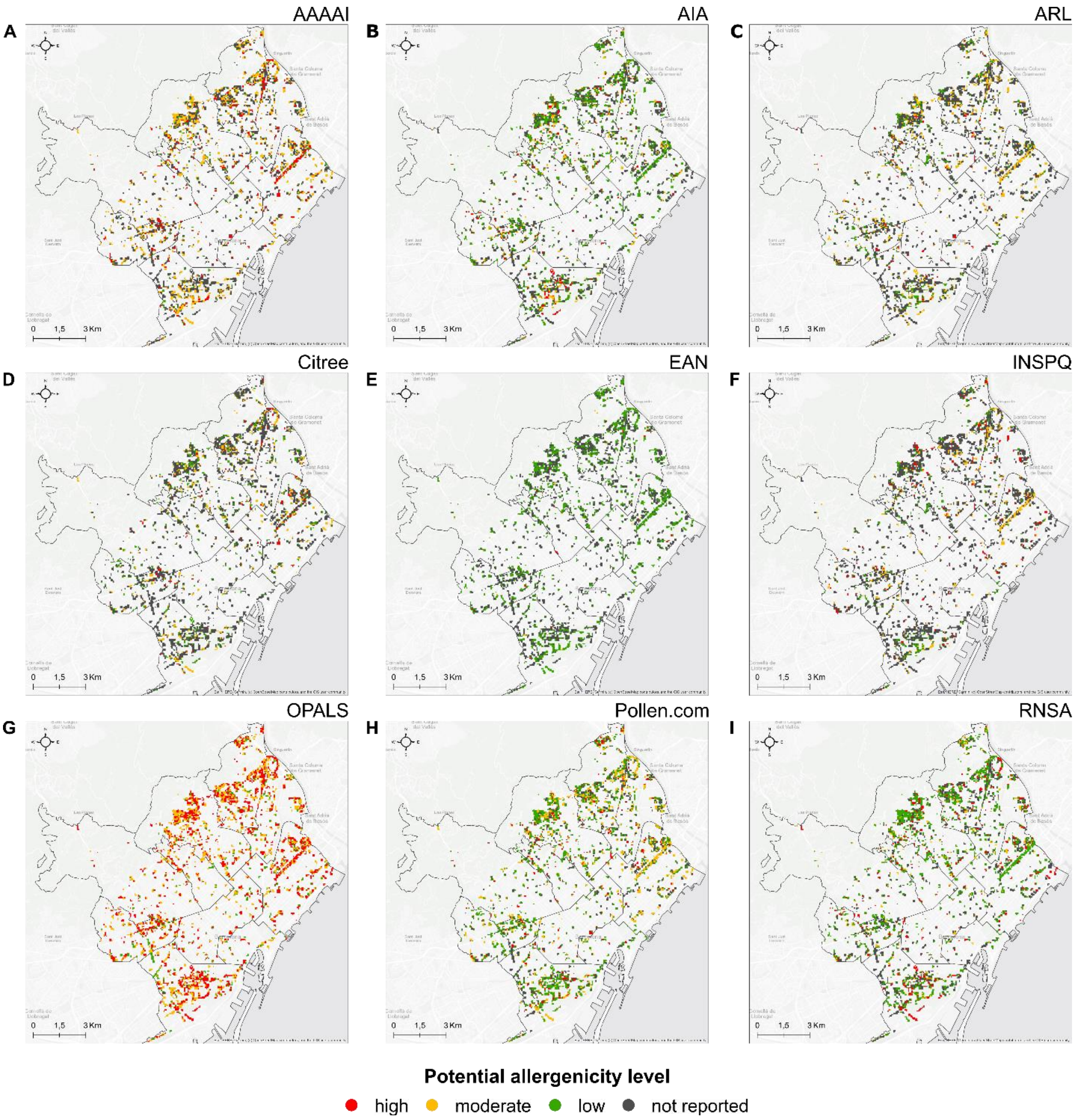

Figure S1. The allergenicity riskscape of Barcelona, Spain, based on the potential allergenicity of its tree species as per the different datasets included in the study. (**A**) the American Academy of Allergy, Asthma & Immunology (AAAAI); (**B**) the vegetation guidance by the Italian Association of Aerobiology (AIA); (**C**) Canada's Aerobiology Research Laboratories (ARL); (**D**) the Citree's library; (**E**) the European Aeroallergen Network (EAN); (**F**) the National Institute of Public Health in Quebec (INSPQ); (**G**) the Ogren Plant Allergy Scale (OPALS); (**H**) the Pollen.com's library; and (**I**) the vegetation guidance by the French aerobiological monitoring network (RNSA). Each dot represents one tree. Maps were created in ArcMap 10.7.1 (http://www.esri.com/).

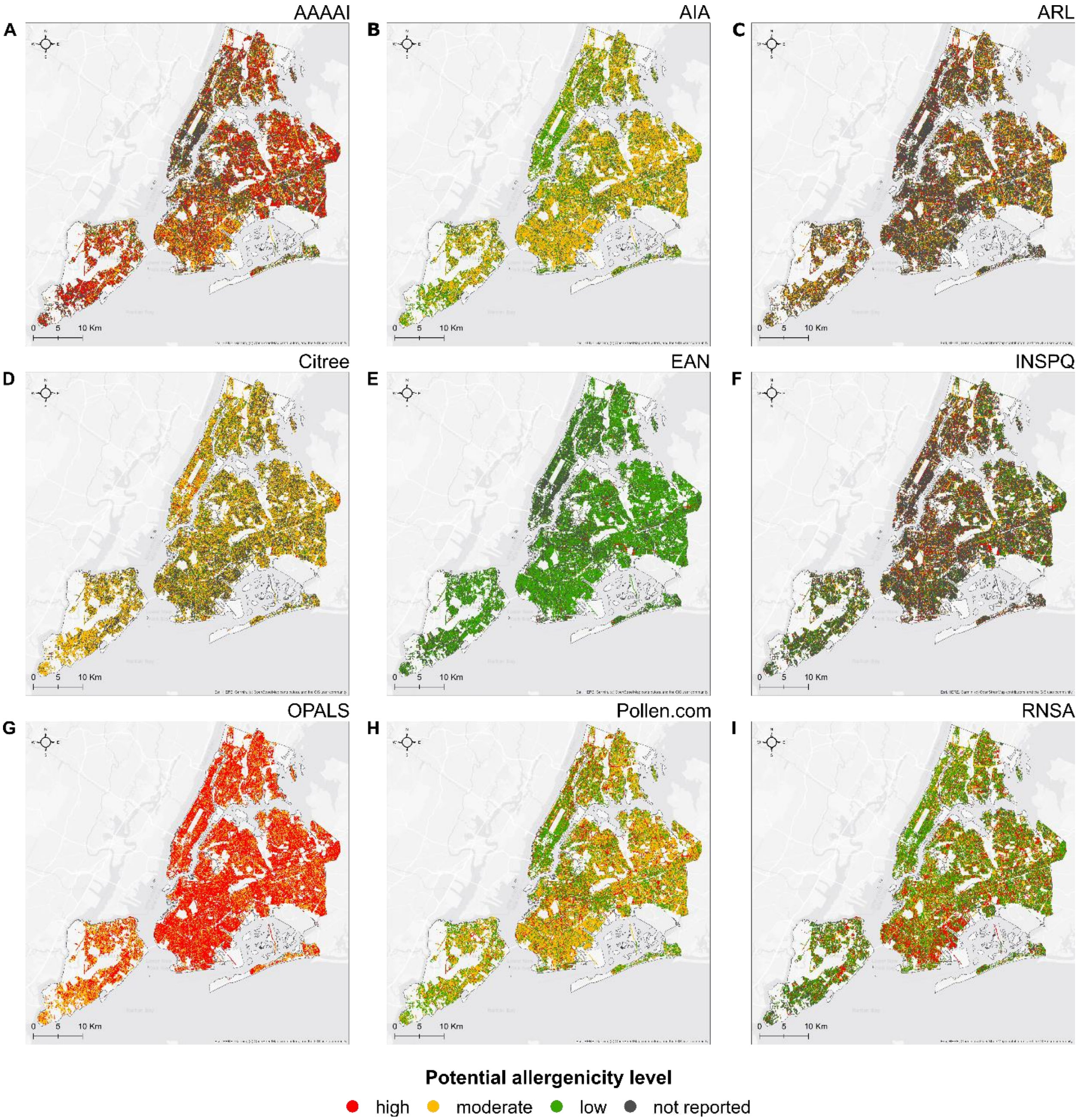

Figure S2. The allergenicity riskscape of New York City, United States, based on the potential allergenicity of its tree species as per the different datasets included in the study. (**A**) the American Academy of Allergy, Asthma & Immunology (AAAAI); (**B**) the vegetation guidance by the Italian Association of Aerobiology (AIA); (**C**) Canada's Aerobiology Research Laboratories (ARL); (**D**) the Citree's library; (**E**) the European Aeroallergen Network (EAN); (**F**) the National Institute of Public Health in Quebec (INSPQ); (**G**) the Ogren Plant Allergy Scale (OPALS); (**H**) the Pollen.com's library; and (**I**) the vegetation guidance by the French aerobiological monitoring network (RNSA). Each dot represents one tree. Maps were created in ArcMap 10.7.1 (http://www.esri.com/).

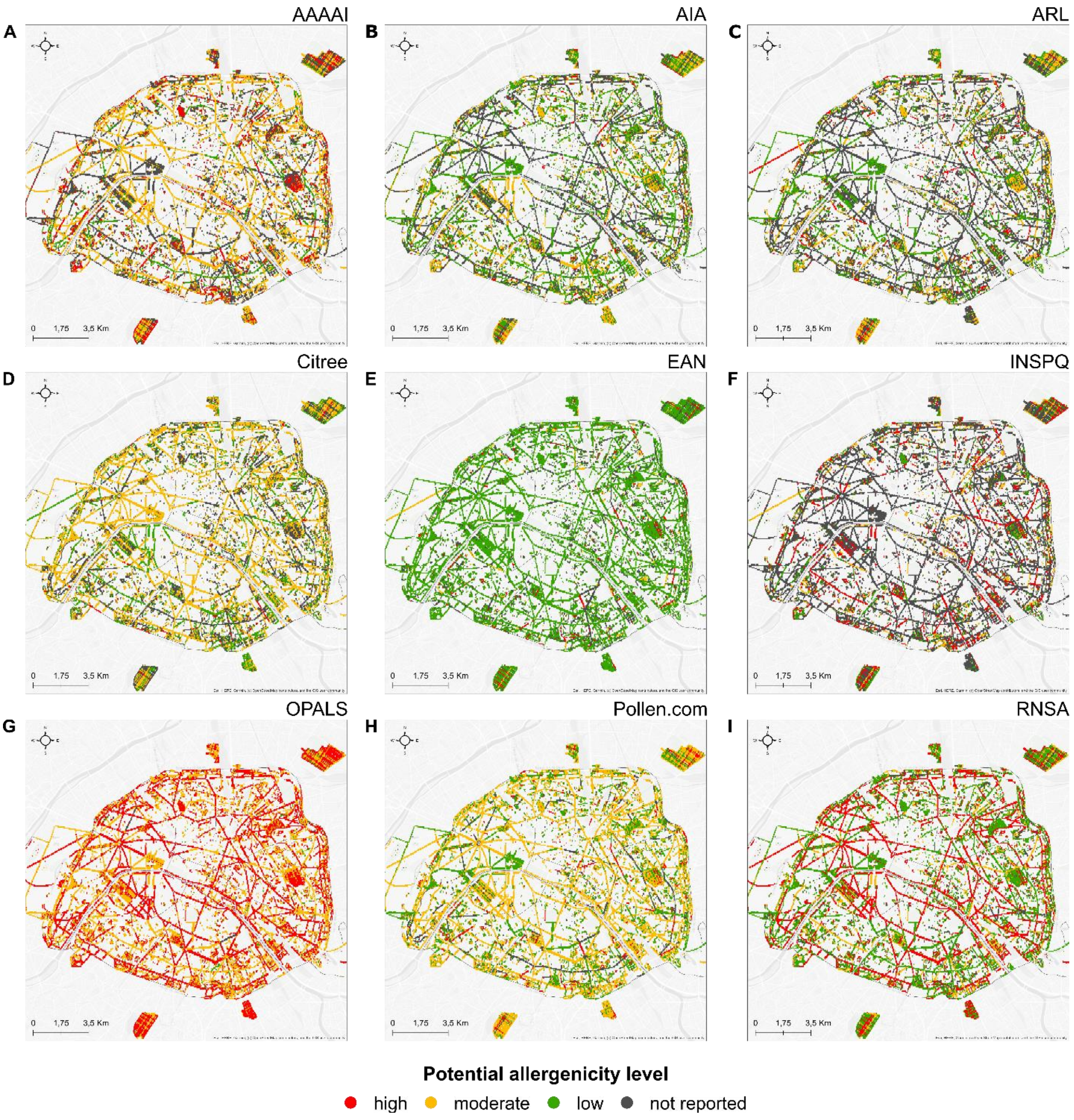

Figure S3. The allergenicity riskscape of Paris, France, based on the potential allergenicity of its tree species as per the different datasets included in the study. (**A**) the American Academy of Allergy, Asthma & Immunology (AAAAI); (**B**) the vegetation guidance by the Italian Association of Aerobiology (AIA); (**C**) Canada's Aerobiology Research Laboratories (ARL); (**D**) the Citree's library; (**E**) the European Aeroallergen Network (EAN); (**F**) the National Institute of Public Health in Quebec (INSPQ); (**G**) the Ogren Plant Allergy Scale (OPALS); (**H**) the Pollen.com's library; and (**I**) the vegetation guidance by the French aerobiological monitoring network (RNSA). Each dot represents one tree. Maps were created in ArcMap 10.7.1 (http://www.esri.com/).

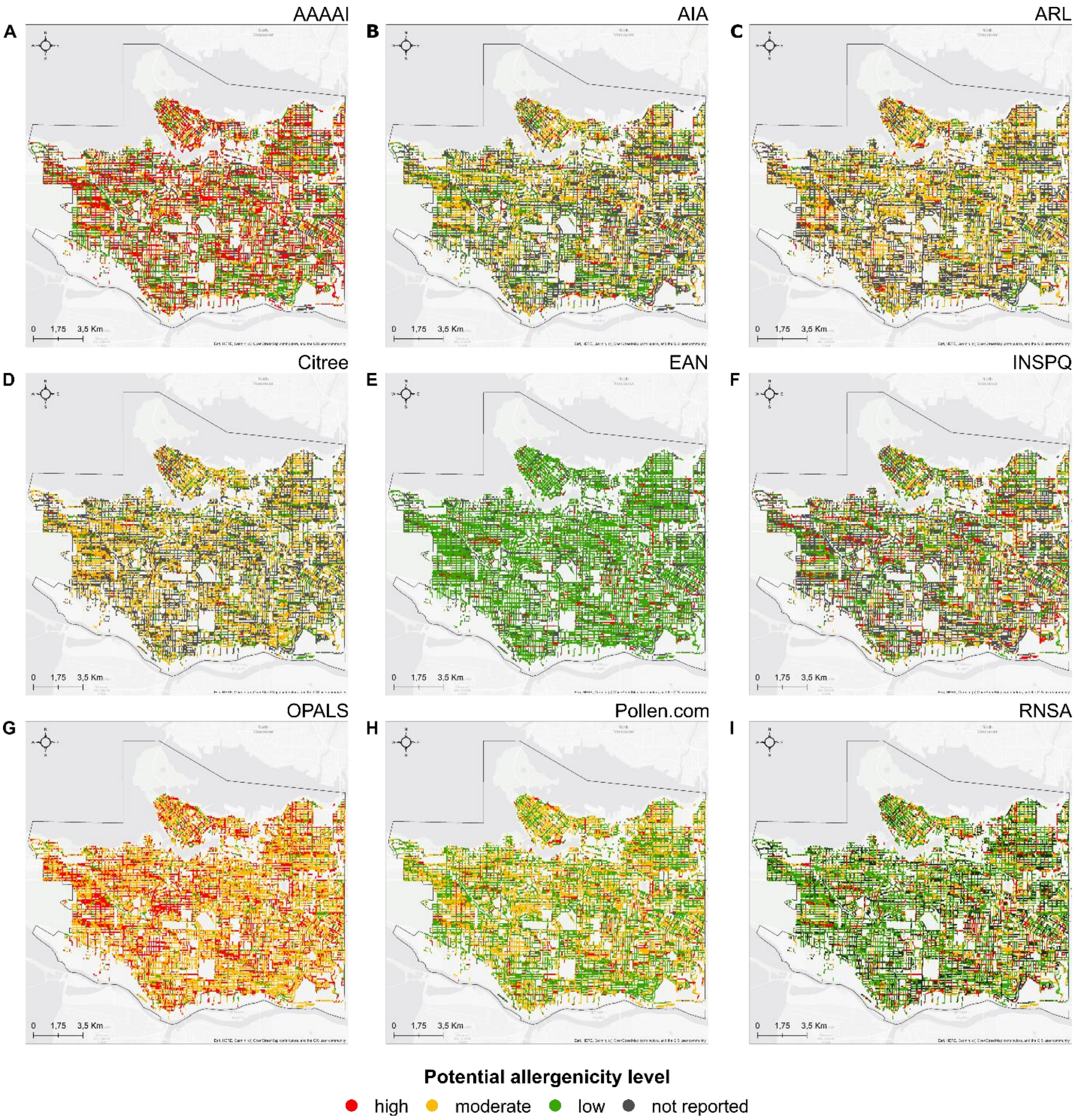

Figure S4. The allergenicity riskscape of Vancouver, Canada, based on the potential allergenicity of its tree species as per the different datasets included in the study. (**A**) the American Academy of Allergy, Asthma & Immunology (AAAAI); (**B**) the vegetation guidance by the Italian Association of Aerobiology (AIA); (**C**) Canada's Aerobiology Research Laboratories (ARL); (**D**) the Citree's library; (**E**) the European Aeroallergen Network (EAN); (**F**) the National Institute of Public Health in Quebec (INSPQ); (**G**) the Ogren Plant Allergy Scale (OPALS); (**H**) the Pollen.com's library; and (**I**) the vegetation guidance by the French aerobiological monitoring network (RNSA). Each dot represents one tree. Maps were created in ArcMap 10.7.1 (http://www.esri.com/).
